# Beneficial effects of a mouthwash containing an antiviral phthalocyanine derivative on the length of hospital stay for COVID-19: randomised trial

**DOI:** 10.1038/s41598-021-99013-5

**Published:** 2021-10-07

**Authors:** Paulo Sérgio da Silva Santos, Bernardo da Fonseca Orcina, Rafael Rahal Guaragna Machado, Fabiano Vieira Vilhena, Lucas Marques da Costa Alves, Mariana Schutzer Ragghianti Zangrando, Rodrigo Cardoso de Oliveira, Mariana Quirino Silveira Soares, Andréa Name Colado Simão, Emilene Cristine Izu Nakamura Pietro, Juliana Pescinelli Garcia Kuroda, Ivanilda Aparecida de Almeida Benjamim, Danielle Bastos Araujo, Sérgio Hiroshi Toma, Lourival Flor, Koiti Araki, Edison Luiz Durigon

**Affiliations:** 1 Bauru School of Dentistry of University of São Paulo, Al. Dr. Octavio Pinheiro Brisolla, 9-75, Vila Universitária, Bauru, São Paulo 17012-901 Brazil; 2 Institute of Biomedical Sciences, University of Sao Paulo, São Paulo, Brazil; 3 TRIALS – Oral Health & Technologies, Bauru, SP Brazil; 4 Hospital Estadual de Bauru, Bauru, Brazil; 5 Faculdade São Leopoldo Mandic, Instituto de Pesquisa São Leopoldo Mandic, Campinas, Brazil; 6 Londrina State University, Paraná, Brazil; 7 Golden Technology Corp, São Paulo, Brazil; 8 Institute of Chemistry, University of Sao Paulo, São Paulo, Brazil

**Keywords:** Microbiology, Health care

## Abstract

The risk of contamination and dissemination by SARS-CoV-2 has a strong link with nasal, oral and pharyngeal cavities. Recently, our research group observed the promising performance of an anionic phthalocyanine derivative (APD) used in a mouthwash protocol without photoexcitation; this protocol improved the general clinical condition of patients infected with SARS-CoV-2. The present two-arm study evaluated in vitro the antiviral activity and cytotoxicity of APD. Additionally, a triple-blind randomized controlled trial was conducted with 41 hospitalized patients who tested positive for COVID-19. All the included patients received World Health Organization standard care hospital treatment (non-intensive care) plus active mouthwash (experimental group AM/n = 20) or nonactive mouthwash (control group NAM/n = 21). The adjunct mouthwash intervention protocol used in both groups consisted one-minute gargling/rinsing / 5 times/day until hospital discharge. Groups were compared considering age, number of comorbidities, duration of symptoms prior admission and length of hospital stay (LOS). The associations between group and sex, age range, presence of comorbidities, admission to Intensive care unit (ICU) and death were also evaluated. The in vitro evaluation demonstrated that APD compound was highly effective for reduction of SARS-CoV-2 viral load in the 1.0 mg/mL (99.96%) to 0.125 mg/mL (92.65%) range without causing cytotoxicity. Regarding the clinical trial, the median LOS of the AM group was significantly shortened (4 days) compared with that of the NAM group (7 days) (p = 0.0314). Additionally, gargling/rinsing with APD was very helpful in reducing the severity of symptoms (no ICU care was needed) compared to not gargling/rinsing with APD (28.6% of the patients in the NAM group needed ICU care, and 50% of this ICU subgroup passed way, p = 0.0207). This study indicated that the mechanical action of the protocol involving mouthwash containing a compound with antiviral effects against SARS-CoV-2 may reduce the symptoms of the patients and the spread of infection. The use of APD in a mouthwash as an adjuvant the hospital COVID-19 treatment presented no contraindication and reduced the hospital stay period.

**Trial registration:** The clinical study was registered at REBEC—Brazilian Clinical Trial Register (RBR-58ftdj).

## Introduction

SARS-CoV-2 was first recognized at the end of 2019, and its outbreak caused a global pandemic that is affecting people all over the world due to it higher contamination rate, spreading capacity and index of lethality than those of previous coronaviruses^1–3^. Patients who test positive for COVID-19 are admitted to hospitals and receive intensive care at unprecedent rate^4^. The patients who are most vulnerable to the development of COVID-19 are elderly people who suffer from comorbidities, such as high blood pressure, obesity, heart diseases, breathing problems and neoplasia^4^. Thus, according to the World Health Organization (WHO), an early and accurate diagnosis is crucial to control the spread of SARS-CoV-2 because a higher viral load is related to more severe disease^5,6^.

It is clear that washing hands, wearing masks, and social distancing are effective measures to fight the pandemic^6,7^. In addition, considering that COVID-19 contagion, evolution and dissemination have strong associations with the mouth^8,9^, gargling with antiseptic mouthwashes has been suggested as an extra preventive measure against COVID-19^7,8,10–17^. However, in the cases of diseases, such as influenza, which are caused by SARS-type viruses, virucidal activity is essential for such measures to be effective^8^.

Phthalocyanines are analogues of synthetic and aromatic planar porphyrin macrocycles consisting of four indol units linked together by nitrogen atoms^18^, and have shown good inactivation of various microbial pathogens^19^. Furthermore, they are dyes awaiting which, with the combination of a sensitizing drug with visible light, will promote the selective destruction of viruses, bacteria and other microorganisms^20^.The potential of phthalocyanines for biological and medical applications has been recognized^20–23^, especially in photodynamic therapy, since phthalocyanines in the excited state can promote the reactive oxygen species generation or redox processes, while no such properties are observed in the absence of light. Recently, our research group observed the promising performance of an anionic phthalocyanine derivative (APD) used in a mouthwash protocol without photoexcitation; this protocol improved the general clinical condition of patients with SARS-CoV-2 infection^14,15^.

Hence, the following study aimed to (1) evaluate the antiviral activity and cytotoxicity of APD in vitro and (2) clinically assess the use of an APD-containing mouthwash in hospitalized patients who tested positive for COVID-19 to reduce the severity of the disease and minimize the LOS.

## Methods

This two-arm study consisted of laboratory experiments to evaluate the antiviral activity and cytotoxicity of the anionic iron tetracarboxyphthalocyanine derivative (APD) (Golden Technology Corp., Brazil) and a triple-blind randomized controlled trial.

### Laboratory studies

All the in vitro experiments were conducted in Biosafety Level (BSL) BSL-2 and BSL-3 facilities at the Institute of Biomedical Sciences, University of São Paulo, Brazil, according to the laboratory biosafety guidance recommended by the WHO for the novel coronavirus (SARS-CoV-2)^4^.

- *Antiviral* and cytotoxic *activity of APD*
  To determine the antiviral activity and cytotoxicity of APD, a 2.0 mg/mL (I) stock solution prepared in sterile distilled water was serially diluted by twofold (1.0 mg/mL to 0.39 × 10^–2^ mg/mL, i.e., 1/2 to 1/512) in Dulbecco’s modified essential medium (DMEM) supplemented with 2.5% fetal bovine serum (FBS) in a 96-well cell culture plate to a final volume of 100 μL per well. The dilutions were made in quadruplicate to determine both virus neutralization and cytotoxicity.
  After dilution, 100 μL of SARS-CoV-2 (*SARS.CoV2/SP02.2020.HIAE. Br)* at 10^3^ TCID_50_/mL (MOI = 0,02) was added to the wells and incubated for 30 min at 37 °C. Then, 150 μL of the mixture (APD plus virus) was transferred to a 96-well cell culture plate previously seeded with 1 × 10^5^ Vero CCL-81 cells/mL and grown to 80–90% confluence. The cells were then incubated at 37 °C in a 5% CO_2_ atmosphere for 72 h^24^.
  The plate was visually evaluated using an optical microscope to determine cell integrity and morphology, and then, samples were collected (in quadruplicate) for RNA extraction and real-time Polymerase Chain Reaction (RT-PCR) for the quantitative detection of the amount of active virus. The cells were fixed with Naphthol Blue Black (Sigma-Aldrich^®^).
- *Nucleic acid extraction and real-time RT-PCR for SARS-CoV-2 RNA detection*
  The extraction of total nucleic acids (RNA and DNA) was carried out using the semiautomated NucliSENS^®^ easyMag^®^ platform (BioMerieux, Lyon, France) following the manufacturer's instructions. The detection of viral RNA was carried out using the AgPath-ID One-Step RT-PCR Kit (Applied Biosystems Inc., EUA) on an ABI 7500 SDS real-time PCR machine (Applied Biosystems, Weiterstadt, Germany) using a published protocol and primers and probes specific for the E^25^ RNA copies/mL were quantified by real-time RT-PCR using a specific in vitro-transcribed RNA quantification standard kindly provided by Christian Drosten, Charité—Universitätsmedizin Berlin, Germany, as described previously^26^.

The antiviral activity was expressed as the percent reduction in the active SARS-CoV-2 virus RNA/mL, calculated according to Eq. (1), after contact with the test specimen compared to the number of virus particles in the positive control.

1 $$ {\text{Reduction }}\left( \% \right) = \left[ {\left( {{\text{B}} - {\text{A}}} \right)/{\text{B}}} \right] \, \times { 1}00. $$

where A and B are the numbers of RNA copies/mL recovered from the supernatant of APD-treated and APD-untreated cells, respectively.

- *Indirect Immunofluorescence (IIF)*

The methodology described here was adapted from Sales-Medina et al.^27^. Briefly, at 72 h.p.i., the plates were fixed for 30 min in 4% paraformaldehyde in 1X PBS (pH 7.4) and subjected to indirect immunofluorescence detection of viral cellular infection. After washing twice with 1X PBS 0.05% Tween 20 (PBST), the plates were blocked with bovine serum albumin (BSA) (3% w/v in 1X PBS; Sigma-Aldrich) for 30 min at room temperature and washed twice with PBST. Convalescent serum from a Brazilian patient with COVID-19 diluted 1:1000 in PBS was used as a primary antibody to detect SARS-CoV-2 in Vero cells. The primary antibodies were incubated for 30 min, and the plates were washed twice with PBST. Subsequently, goat anti-human IgG labeled with Alexa 488 (Thermo Scientific) diluted to 4 μg/mL in PBS was used as the secondary antibody, and the cells were incubated for 30 min with 5 μg/mL 4′,6-diamidine-2′-phenylindole dihydrochloride (DAPI, Sigma-Aldrich) in PBS to stain the nucleic DNA. The plates were washed twice with PBST and imaged in an Operetta High Content Imaging System (Perkin Elmer) using a 20 × magnification objective. Five images were acquired per well and analyzed using Harmony software (Perkin Elmer), version 3.5.2. Image analysis consisted of identifying and counting the Vero E6 cells based on the nuclear segmentation, viral infection, and cytoplasmic staining detected by the immunofluorescence assay^27^.

### Clinical trial design and oversight

This triple-blind randomized controlled trial was conducted in accordance with the principles of the Declaration of Helsinki and ethical standards of human experimentation with the approval of the Human Research Ethics Committee of Bauru School of Dentistry of the University of Sao Paulo, Brazil (CAAE 34,070,620.6.0000.5417). This clinical study was also registered at REBEC—Brazilian Clinical Trial Register (RBR-58ftdj) in 10/28/2020. The study complied with the Consort 2010 checklist of information to include when reporting a randomized trial. This study was carried out as a controlled trial from 10th August to 4^th^ November 2020 at Bauru State Hospital, Bauru, with hospitalized patients who tested positive for SARS-CoV-2. All the participants received the World Health Organization standard care hospital treatment (world medical protocol—antibiotics, corticoids and anticoagulants)^6^ plus one of the two mouthwash interventions (active and nonactive mouthwashes). Based on previous studies, APD antimicrobial compound-containing mouthwash was chosen as the active mouthwash (AM)^14,15,28^ for comparison with a nonactive mouthwash (NAM) negative control. Both mouthwashes were produced with exactly the same formula (color, flavor, other ingredients) except for the presence or absence of the active ingredient. Once the mouthwash intervention was given to patients receiving medical treatment for COVID-19, the use of a negative control mouthwash was necessary. The active compound concentration used was between the 1:8 and 1:16 titer according to the in vitro arm of the study. All the oral care materials used during the study (mouthwashes, toothbrushes, toothpaste and dental floss) were produced by Rabbit Corp, Brazil.

### Patient inclusion and exclusion criteria

The inclusion criteria were patients aged 18 years or older, who were hospitalized less than 7 days after the onset of severe acute respiratory syndrome (SARS), who were suspected and who were confirmed positive for SARS-CoV-2 by RT-PCR test, admitted to the hospital with a mild or moderate clinical condition, classified according WHO recommendations^6^ with no need for intensive care unit (ICU). To be enrolled in the study, each participant read and signed the informed consent form after understanding the risks and objectives of the study. The exclusion criteria included patients who had contraindications to using mouthwash due to medical reasons or the inability to gargle and spit.

### Randomization and masking

Sample randomization was performed as follows: the mouthwash bottles (AM and NAM) were placed in a closed package with consecutive numbers according to the patients selected. An EXCEL^®^ database was created from these numbered packages and used for randomization. After randomization, packages with mouthwash bottles and oral care kits were delivered to the hospitalized patients. This study was considered triple-blind because the patients, the examiner and the statistician were blinded to the treatment groups.

### Mouthwash intervention

The eligible participants were randomly assigned to one of the following groups: AM or NAM. The patients were instructed to use 5 mL of the mouthwash and to switch between gargling/rinsing for 1 min up to 5 times a day: upon awakening, after breakfast, after lunch, after dinner and before bedtime^14,15^. Each patient followed this adjunctive therapeutic protocol until the outcome associated with COVID-19 medicine treatment was recorded.

### Outcome measures

The primary outcome was the time to clinical improvement, defined as the length of stay in the hospital (patients did not need oxygen therapy support for more than 24 h and no longer showed any symptoms). Secondary outcomes were clinical evolution, need for care in the ICU and death. The criteria for the transfer of a patient to the ICU was the presence of respiratory effort requiring the use of O_2_ above 8.0 L/min, which is the initial graduation of nonremovable masks. The mouthwash protocol was also verified regarding the conditions of use and side effects^14,15^.

### Statistical analysis

Statistical analysis was conducted using R (R Core Team) and SAS® software version 3.8 (SAS Institute Inc). Descriptive and exploratory data analyses were performed. A Mann–Whitney U nonparametric test was used to compare the groups regarding age, number of comorbidities, and duration of symptoms prior to hospitalization. The frequency of admission to intensive care and deaths were compared between groups using Fisher’s exact test. The time to clinical improvement is presented by a Kaplan–Meier plot and was compared using a Cox regression model, with an estimate of the hazard ratio association measure and 95% confidence interval. Since a significant difference was observed between groups regarding the median age, the survival analysis was adjusted for the patient's age.

The sample of 41 patients included in this investigation provided a test power of 0.80 for a minimum detectable hazard ratio of 2.5, with α = 0.05, considering the follow-up time of 22 days and median hospitalization time in the control group of 7 days.

### Ethics approval and consent to participate

This research was approved of the Human Research Ethics Committee of Bauru School of Dentistry of the University of Sao Paulo, Brazil (CAAE 34070620.6.0000.5417).

## Results

### In vitro cytotoxicity and antiviral activity

**Figure 1:**
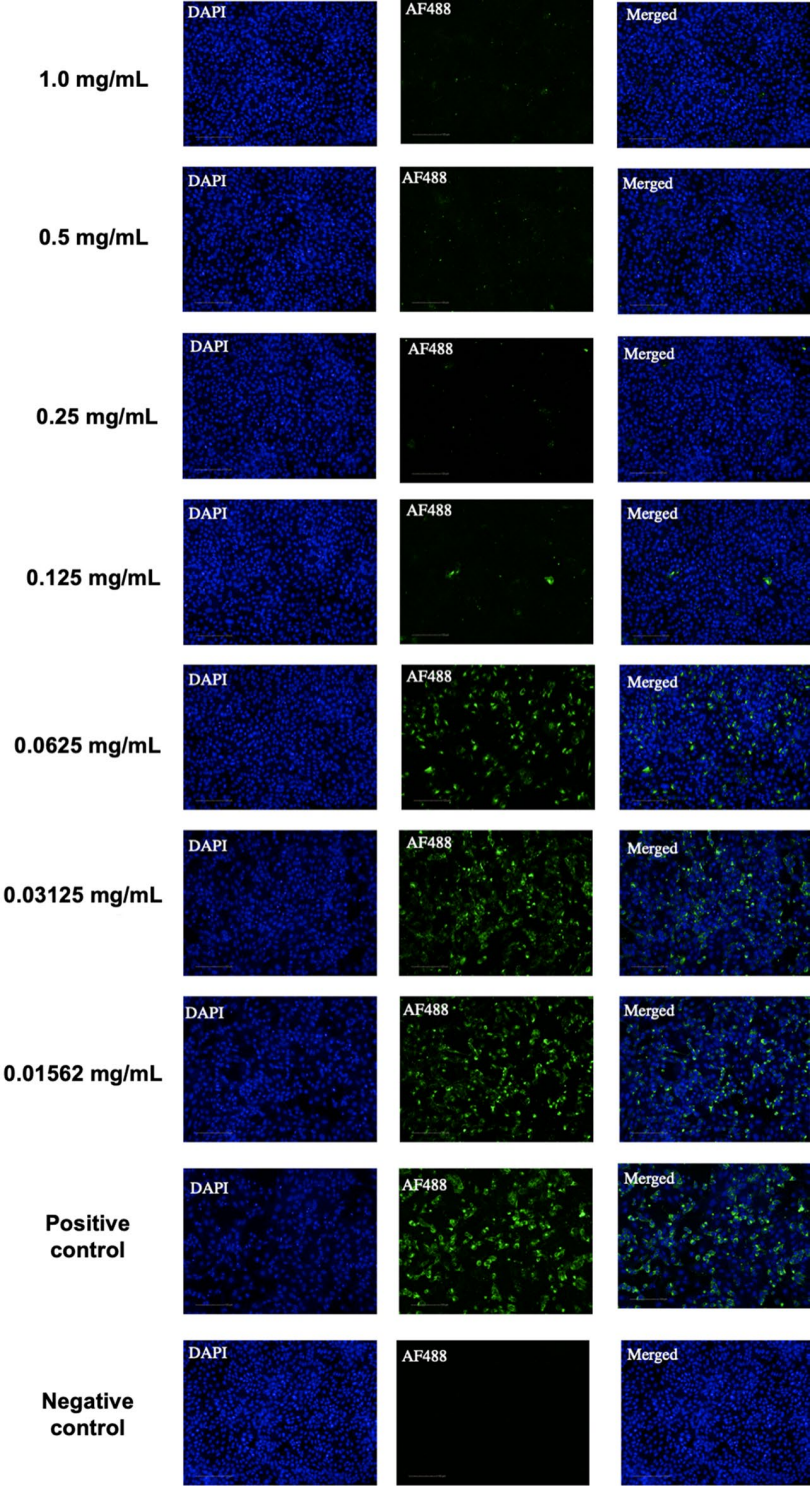
Indirect immunofluorescence (IIF) *assay for the detection of SARS-CoV-2-infected cells.* Representative images of the noncytotoxic concentrations (1.0 mg/mL up to 0.156 mg/mL) of APD observed with a 20 × objective. A mixture of MERS-CoV-infected and noninfected Vero cells was stained with convalescent serum monoclonal antibodies, followed by incubation with the Alexa488-conjugated goat anti-human IgG antibodies (green). Cells were counterstained with DAPI to stain the nuclei (blue). Positive (infected nontreated cells) and negative (noninfected cells) controls are shown at the bottom of the image. Images were taken using the Operetta High Content Imaging System (Perkin Elmer). Scale bar, 100 µm.

In the optical microscopy observations, APD demonstrated cytotoxicity only at the initial dilution (2.0 mg/mL, the most concentrated stock solution) since Vero *CCL-81* cell monolayer integrity was observed after treatment with all the other dilutions. The integrity of the cell monolayer was observed under a microscope after treatment with APD at dilutions ranging from 1:2 (1.0 mg/mL) to 1:64 (1.56 × 10^–2^ mg/mL); these results were confirmed by cell fixation and staining with Naphthol Blue Black. The data for both the cell cytotoxicity and antiviral activity of APD were also confirmed by indirect immunofluorescence (IIF) (Fig. 1 and supplementary material). The real-time RT-PCR results showed a significant reduction in viral load when compared to the positive control at the 1:2 (99.96%), 1:4 (99.88%), 1:8 (99.84%) and 1:16 (92.65%) titers, whereas partial virus neutralization was observed at the 1:32 (77.42%) and 1:64 (11.06%) titers. No virus neutralization was observed below the 1:128 titer.

### Clinical trial

#### Patients

**Figure 2:**
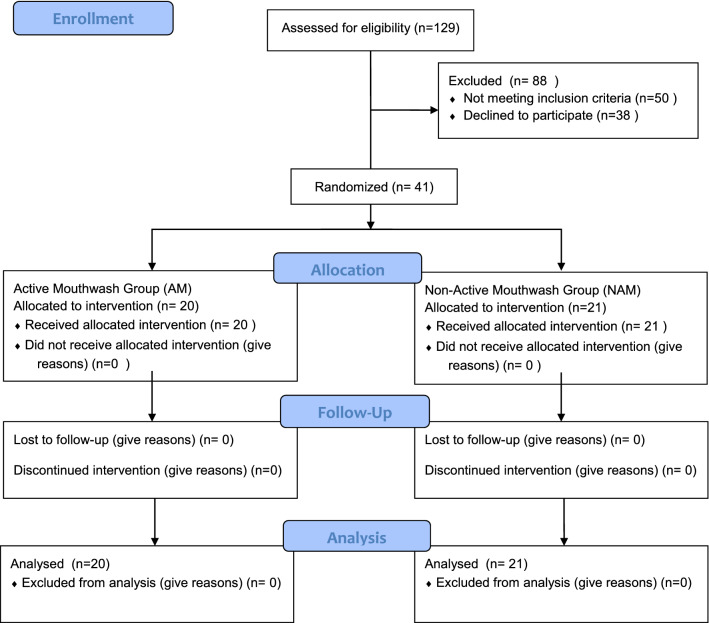
CONSORT 2010 Flow Diagram with randomly divided groups AM and NAM.

According to CONSORT, 129 patients with suspected COVID-19 and admitted to the hospital were recruited for the study. After removing patients based on their oropharynx RT-PCR results, the inclusion and exclusion criteria (n = 50), and declining to participate or discontinuing the intervention (n = 38), 41 patients positive for COVID-19 were finally eligible for this study. These patients were randomly divided to the AM group (n = 20) or the NAM group (n = 21) (Fig. 2).

The median age of the patients was significantly higher in the AM group than in the NAM group (p = 0.0069). The participants in the NAM group were 48.4 years old on average, ranging from 27 to 70 years old, and those in the experimental group were 59.1 years old on average, ranging from 32 to 78 years old (Table 1). Additionally, 19% and 50% of the patients in the NAM and AM groups, respectively, were aged over 60 years (p = 0.0367). There was no significant difference between the groups regarding sex, presence of comorbidities, number of comorbidities, duration of symptoms prior to hospitalization (p > 0.05).

**Table 1:** Demographic and clinical data of both groups of patients: active mouthwash (AM) and non-active mouthwash (NAM) groups.

	Group				p-value
	NAM		AM		
	Mean (SD)	Median (min–max)	mean (SD)	Median (min–max)	
Age (years)	48.4 (11.4)	47.0 (27.0–70.0)	59.1 (13.0)	59.0 (32.0–78.0)	^1^0.0069
Number of comorbidities	1.4 (1.4)	1.0 (0.0–4.0)	1.6 (1.3)	1.5 (0.0–5.0)	^1^0.6481
Duration of symptoms prior to hospitalization (days)	4.3 (2.1)	4.0 (1.0–9.0)	5.3 (1.9)	6.0 (1.0–9.0)	^1^0.1238

	n	%	n	%	
Males	13	61.9%	13	65.0%	^2^0.8370
Age > 60 y	4	19.0%	10	50.0%	^2^0.0367
Presence of comorbidities	14	66.7%	16	80.0%	^2^0.3355

^1^Mann Whitney-U; ^2^ Chi-square; *SD* standard deviation.

Regarding O_2_ saturation, in Group AM we found 8 (40%) patients with SaO_2_ > 95%, 10 (50%) with SaO_2_ between 90–94%, 1 (5%) with SaO_2_ < 90%, and 1 patient not informed in the medical chart. In the NAM group we found 5 (23.8%) patients with SaO_2_ > 95%, 15 (71.4%) with SaO_2_ between 90–94%, and 1 (4.8%) with SaO_2_ < 90%. These patients were receiving O_2_ support respectively, in the AM group with room air 8 (40%), 1 to 3 l/min of O_2_—8 (40%), 4 to 5 l/min of O_2_—2 (10%) and more than 5 l/min of O_2_—1 (5%). In the NAM group with room air 5 (23.8%), 1 to 3 l/min of O_2_—11 (52.4%), 4 to 5 l/min of O_2_—3 (14.3%) and more than 5 l/min of O_2_—2 (9.5%).

### Primary outcome

**Figure 3:**
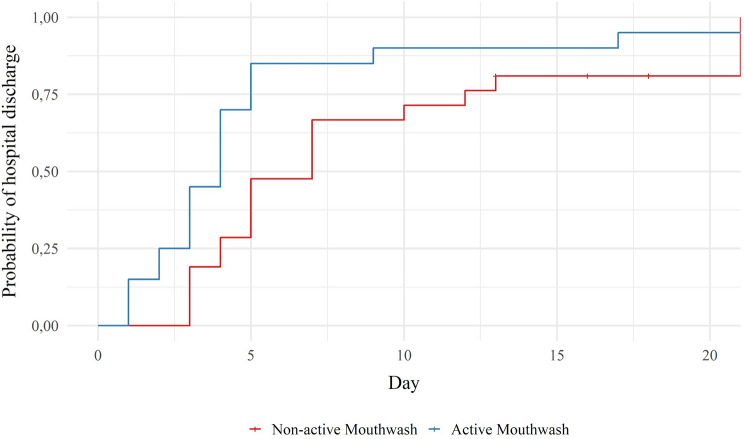
Probability of hospital discharge over time (days) according to each group.

Table 2 and Fig. 3 show that there was a significant difference between the two groups regarding the occurrence of hospital discharge over time (p < 0.05). As there was a significant difference between the groups in terms of age, the survival analysis was performed with adjustment for age. The median LOS, that is, the time at which 50% of patients were discharged, for the NAM group was 7 days. In the AM group, this time was 4 days. It is also noted that the time for 75% of the patients in the NAM group to be discharged was 12 days, whereas in the AM group, this time was 5 days. The hazard ratio for hospital discharge was 2.16 (95% CI 1.07–4.34).

**Table 2:** Duration of hospitalization between groups.

	Data distribution	Time (days)
NAM	75% (IC95%)	12.0 (7.0–21.0)
	50% (IC95%)	7.0 (4.0–10.0)
	25% (IC95%)	4.0 (3.0–5.0)
	Mean (SD)	8.9 (1.4)
AM	75% (IC95%)	5.0 (4.0–17.0)
	50% (IC95%)	4.0 (2.0–5.0)
	25% (IC95%)	2.5 (1.0–3.0)
	Mean (SD)	5.10 (1.19)

Hazard ratio = 2.16 (IC 95%: 1.07–4.34), p = 0.0314.

*AM* Active Mouthwash, *NAM* Non-Active Mouthwash groups, *SD* Standard deviation.

### Secondary outcomes

There was a significant association between the need for admission to the ICU and the group to which patients belonged (p < 0.05). In the NAM group, 6 (28.6%) patients needed intensive care during hospitalization, while in the AM group, none of the patients required ICU admission (p = 0.02). Three patients (14.3%) in the NAM group and none in the experimental group died during hospitalization (p = 0.23) (Table 3).

**Table 3:** Need of Intensive Care Unit (ICU) and deaths in NAM and AM groups.

	Group				p-value
	NAM		AM		
	n	%	n	%	
Need of ICU	6	28.6%	0	0.0%	^a^0.0207
Death	3	14.3	0	0.0%	0.2317

^a^Fisher’s exact test. *NAM* Non active mouthwash, *AM* Active mouthwash.

### Safety outcomes

The use of the gargle/rinse protocol was possible and tolerable with no side effects. All the participants reported a pleasant, sweet taste after using both AM and NAM.

## Discussion

The in vitro evaluation demonstrated promising antiviral action with the absence of cytotoxic effects in a range of concentrations of APD. The APD concentration used in the AM in this clinical study confirmed an antiviral efficacy between 92 and 99%, without evidence of cytotoxicity. The AM group presented a significant reduction in the length of hospitalization and the additional benefit of less severe symptoms. Moreover, according to the safety outcomes, no side effects were reported by the patients from the AM and NAM groups regarding the gargle/rinse mouthwash protocol.

Data regarding the mean time of hospitalization due to COVID-19 vary between geographic areas, but 14 days was found to be a reliable LOS time for patients in hospitals in China^29^. In a recent study^30^ was described a median hospital stay period of 12 days in Brazil, which decreased to 6 to 7 days, depending on the concentration of propolis intervention used by the patients. In the present study, a promising result was found when the median LOS further decreased to 4 days in the AM group, which differed significantly from the NAM group, which had an average LOS of 7 days. On the other hand, it is possible to hypothesize that NAM itself was effective in reducing LOS when compared to previous studies^29,30^. This result is probably due to mechanical hygiene of the oral and oropharynx cavities. There is in fact evidence of the effectiveness of the gargle/rinse protocol since local action is associated the regions that are intimately associated with the development of COVID-19 and play an important role in the defense of the host^7,10,31^. In this sense, mechanical hygiene reduces the superficial viral load of the oropharynx and oral mucosa and could prevent upper respiratory tract infections^32^. Other authors^33^ reported the prevention of respiratory infections by gargling povidone-iodine, where gargling more than four times daily was considered effective in preventing the adherence of pathogenic bacteria to the upper respiratory tract. In our case, however, we believe this mechanical action was just an adjuvant that potentialized the effect of APD in reducing the SARS-CoV-2 load, which was clinically demonstrated by the great reduction in LOS. Recent studies have shown that a higher viral load is the key factor in the severity of the disease and in worse prognosis^5,34^. For this reason, decreasing the viral load is crucial to restrain the development of the disease, thus preventing the most serious symptoms. The virus enters the cell through the connection between the virus spike and the ACE2 receptor, which can be abundantly found in the salivary glands. Therefore, some recommendations have suggested the importance of controlling and reducing the viral load in the oral and oropharynx cavity by using an antiviral mouthwash^8–11,13,14,35^.

Several studies have demonstrated the antimicrobial (antiviral) and anti-inflammatory properties of phthalocyanines^20–23^ after photoexcitation. However, in our case, virucidal activity was observed in the dark and with an iron phthalocyanine known to have poor photochemical properties compared to the respective zinc complexes^36^, indicating different mechanisms of action.

Considering that some known technologies may exhibit an effective antagonistic action against SARS-CoV-2, a virtual screening based on molecular dynamics simulations and the interaction free energies of 8.770 FDA drugs extracted from the DrugBank database (https://www.drugbank.ca/) was carried out by a team of researchers^21^ which recently proposed phthalocyanine, hypericin, TMC-647055 and quarfloxin derivatives as the potentially most effective drugs for the treatment of COVID-19. All four molecules are known to have antiviral properties, but the results indicated that their high affinity for the inner cavity of the spike glycoprotein in the prefusion conformation could block the HR1 region, thus preventing the conformational changes necessary for SARS-CoV-2 entry into target cells. In other words, these molecules are potential spike glycoprotein fusion inhibitors able to prevent docking and thus infection of host cells by coronavirus.

The main interaction responsible for this fusion inhibition effect of phthalocyanine is hydrophobic, but very significant polar interactions, including hydrogen bonds, seem to be responsible for this effect of hypericin, the second most potent molecule. Accordingly, a stronger interaction and affinity of APD with the inner spike glycoprotein cavity in the prefusion conformation is expected since APD has a similar size and carboxylic acid groups at the periphery that are prone to hydrogen bond interactions. Consequently, the antiviral properties of APD could be due to its antagonistic effects on SARS-CoV-2, but APD is also known for its capacity to interact and activate oxygen molecules in the air, inducing a very localized production of activated oxygen molecules. These molecules are able to cause oxidative stress/damage to microorganisms, such as the coronavirus, leading to their inactivation. This hypothesis is confirmed by the effect of noncytotoxic, low concentrations (1.0 mg/mL up to 0.0156 mg/mL) in reducing in the active viral load after proliferation in Vero *CCL-81* cells as observed by RT-PCR and by the *HTCI immunofluorescence-based assay, which showed the rapid cell nucleus localization based on DAPI staining * (Fig. 1). Thus, the high effectiveness of APD can probably be explained by a dual mode of action, namely, by blocking the HR1 region and by promoting oxidative damage leading to inactivation of the virus, thus making APD a very promising molecule to reduce the SARS-CoV-2 viral load^13^. Thus, it can be suggested that a mouthwash containing APD can help improve the response of the organism to COVID-19 infection. Once the positive response of the phthalocyanine derivative-containing mouthwash in the present study and in previous studies^14,15^ can be observed, the authors also speculate that other properties of phthalocyanines will be identified. In this sense, the possibilities of the local effect of APD associated with the control of secondary infections (antimicrobial activity), the anti-inflammatory effect, and the modulatory effect on the immune response are not excluded^20,21,37,38^.

Although the COVID-19 distribution patterns in hospitals vary from one country to another^5,6,29^, in the present study, there were no significant changes when comparing the AM and NAM groups in terms of sex, presence of comorbidities, number of comorbidities, or duration of symptoms prior to hospitalization.

The dynamics of the disease and the hospital environment, medications, stresses due to the pandemic, and sample size are some possible adverse interferences and limitations. Thus, the interpretation and generalization of the results should be performed with reservation. Nevertheless, the clinical trial clearly showed significant differences between the AM and NAM groups. In addition, the way the randomized study was carried out makes it unique since it created 2 nonhomogeneous groups. According to the literature^39^, elderly patients who were more at risk should have taken longer to recover than younger patients. However, this was not the case, as the elderly patients in the AM group showed similar rates of recovery compared with the patients in different age groups. As a consequence, not only was the LOS decreased in this group of patients (AM) but also no intensive therapy was necessary, indicating that there was no tendency of progression into more severe prognostics. In addition, the probability of discharge (event) among participants in the AM group was twice the probability of discharge among participants in the NAM group at any point in time. Conversely, 28.6% of the patients in the NAM group needed to be admitted to the ICU, and half of this subgroup died. Further investigations are encouraged to confirm these results in larger populations.

The outstanding results achieved in the AM group suggest that mouthwash in addition to other medications can be useful in the strategic planning of COVID-19 treatment by the World Health Organization^6^.

## Conclusion

The APD compound was demonstrated to be highly effective in reducing the SARS-CoV-2 viral load in vitro and to exhibit no cytotoxicity in the 1.0 mg/mL to 6.25 × 10^–2^ mg/mL range. Such a result was also confirmed in a clinical trial where gargling and rinsing five times a day was very helpful in reducing the hospital LOS for patients diagnosed with COVID-19. Further investigation is needed to elucidate this mechanism.

## Supplementary Information

Supplementary Information.

## Data Availability

The datasets generated and analysed during the current study are available from the corresponding author on reasonable request.

## Abbreviations

APD: Anionic phthalocyanine derivative
WHO: World Health Organization
AM: Active mouthwash
NAM: Nonactive mouthwash
LOS: Length of hospital stay
ICU: Intensive care unit
DMEM: Dulbecco’s modified essential medium
FBS: Fetal bovine serum
BSA: Bovine serum albumin
REBEC: Brazilian clinical trial register
IIF: Indirect immunofluorescence
ACE-2: Angiotensin-converting enzyme 2
