# Supplementary material for: Beneficial effects of a mouthwash containing an antiviral phthalocyanine derivative on the length of hospital stay for COVID-19: randomised trial
Source: Sci Rep. 2021 Oct 7;11:19937. doi: 10.1038/s41598-021-99013-5 (PMC8497631; doi:10.1038/s41598-021-99013-5)
Supplement: Supplementary file 1 — Supplementary Information. [file 41598_2021_99013_MOESM1_ESM.docx]

**Supplementary Information**

**Figure S1:** Scheme depicting the map of cytotoxicity and SARS-CoV-2 virus neutralization assays at factor 2 dilution of PD carried out in a 96 wells cell culture plate using Vero CCL-81 cells. I = Initial Dilution (2.0 mg/mL), C+ = positive control (virus), C- = negative control (DMEM suppl. 2.5% FBS)


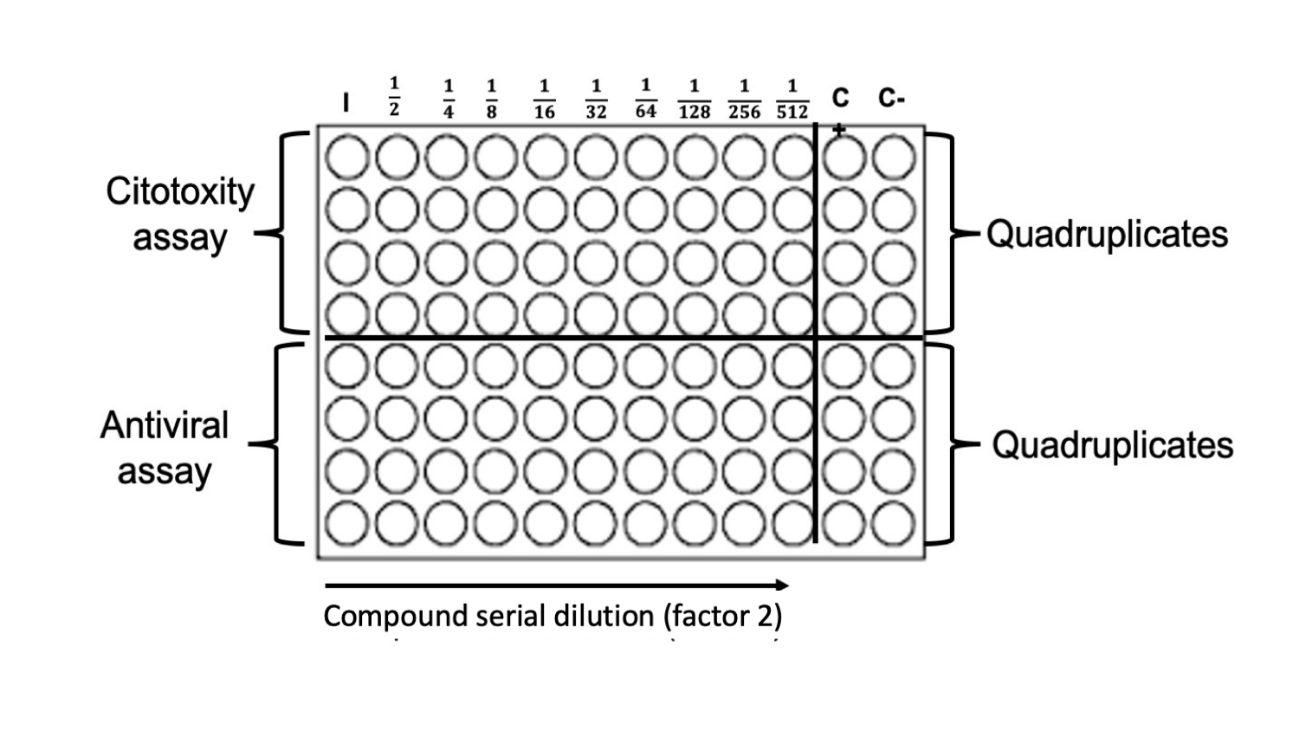


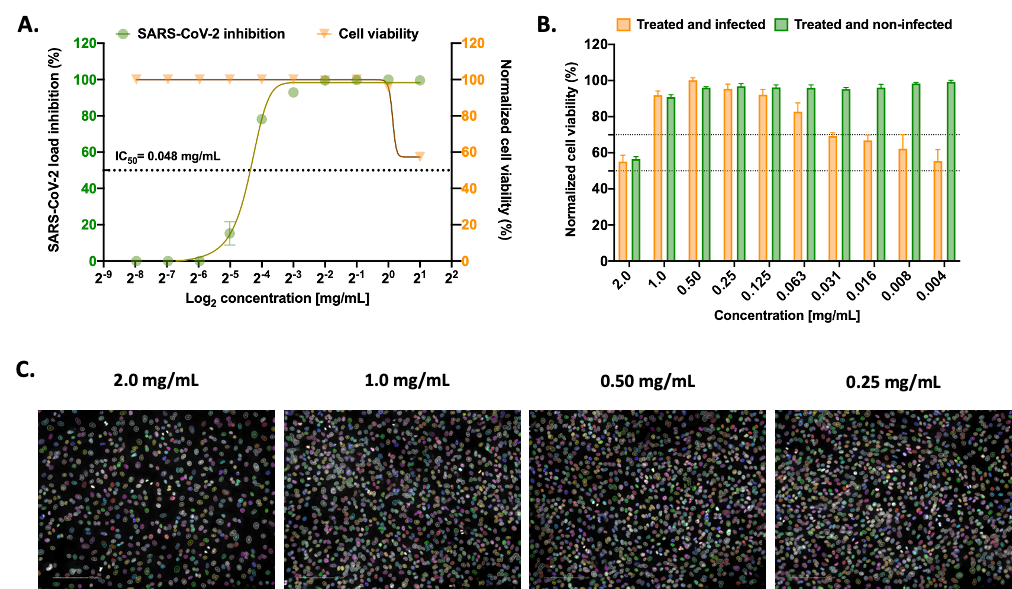


*Fig. S2 (duas colunas): Antiviral activity of APD against SARS-CoV-2 in in vitro assay.  A. The left Y-axis of the graph represents percentage inhibition of virus load in the cellular supernatant. Vero CCL-81 cells were significantly impaired in the presence of 2.0 mg/mL of PD, but did not show citotoxity in all other concentrations. Cells were treated with 8 concentrations of APD (2.0 mg/mL to 0.004 mg/mL). B. High-throughput cellular imaging (HTCI) assay for cell viability quantification 72 hours post treatment and/or infection. Cell viability was normalized to untreated cells. Viability below 70% (cell death more than 30%) was considered evidence of cytotoxicity. Error bars represent the mean ± SEM of two independent experiments carried out in quadruplicate. C. Representative images of Vero cells treated with 2.0, 1.0, 0.50 and 0.25 mg/mL of PD from the HTCI immunofluorescence-based assay showing automated cell nuclei localization based on DAPI staining.*
